# Molecular profiling of pediatric meningiomas shows tumor characteristics distinct from adult meningiomas

**DOI:** 10.1007/s00401-021-02351-x

**Published:** 2021-09-08

**Authors:** Elmar Kirches, Felix Sahm, Andrey Korshunov, Christina Bluecher, Natalie Waldt, Siegfried Kropf, Daniel Schrimpf, Philipp Sievers, Damian Stichel, Ulrich Schüller, Jens Schittenhelm, Markus J. Riemenschneider, Matthias A. Karajannis, Arie Perry, Torsten Pietsch, Svenja Boekhoff, David Capper, Katja Beck, Nagarajan Paramasivam, Matthias Schlesner, Priscilla K. Brastianos, Hermann L. Müller, Stefan M. Pfister, Christian Mawrin

**Affiliations:** 1 Department of Neuropathology, Otto-von-Guericke University Magdeburg, Magdeburg, Germany; 2 Biometry and Medical Informatics, Otto-von-Guericke University Magdeburg, Magdeburg, Germany; 3 Department of Neuropathology, University Hospital Heidelberg, Heidelberg, Germany; 4 Department of Neuropathology, University Hospital Hamburg, Hamburg, Germany; 5 Department of Pediatric Hematology and Oncology, Research Institute Children’s Cancer Center Hamburg, University Hospital Hamburg, Hamburg, Germany; 6 Department of Neuropathology, University Hospital Tuebingen, Tuebingen, Germany; 7 Department of Neuropathology, University Hospital Regensburg, Regensburg, Germany; 8 Department of Neuropathology, University Hospital Bonn, Bonn, Germany; 9 Department of Pediatric Oncology, University Children’s Hospital, Oldenburg, Germany; 10 Department of Neuropathology, Berlin Institute of Health, Charité-Universitätsmedizin Berlin, Freie Universität Berlin, Humboldt-Universität Zu Berlin, 10117 Berlin, Germany; 11 German Cancer Consortium (DKTK), German Cancer Research Center (DKFZ), Partner Site Berlin, Heidelberg, Germany; 12 Bioinformatics and Omics Data Analytics (B240), German Cancer Research Center (DKFZ), Heidelberg, Germany; 13 KiTZ, DKFZ and DKTK, Hopp Children’s Cancer Center Heidelberg, Heidelberg, Germany; 14 Division of Pediatric Neurooncology, Department of Pediatric Hematology and Oncology, Heidelberg University Hospital, Heidelberg, Germany; 15 Department of Pediatric Hematology/Oncology, NYU Langone Medical Center, New York, NY USA; 16 University of California, San Francisco, USA; 17 Massachusetts General Hospital, Boston, USA; 18 Present Address: Pediatric Neuro-Oncology Service, Department of Pediatrics, Memorial Sloan Kettering Cancer Center, New York, NY USA; 19 Center for Behavioral Brain Sciences (CBBS), Magdeburg, Germany; 20 Present Address: Biomedical Informatics, Data Mining and Data Analytics, Faculty of Applied Computer Science and Medical Faculty, University of Augsburg, Augsburg, Germany

**Keywords:** Meningioma, Methylation profile, Targeted sequencing, NF2

## Abstract

In contrast to adults, meningiomas are uncommon tumors in childhood and adolescence. Whether adult and pediatric meningiomas differ on a molecular level is unclear. Here we report detailed genomic analyses of 37 pediatric meningiomas by sequencing and DNA methylation profiling. Histologically, the series was dominated by meningioma subtypes with aggressive behavior, with 70% of patients suffering from WHO grade II or III meningiomas. The most frequent cytogenetic aberrations were loss of chromosomes 22 (23/37 [62%]), 1 (9/37 [24%]), 18 (7/37 [19%]), and 14 (5/37 [14%]). Tumors with *NF2* alterations exhibited overall increased chromosomal instability. Unsupervised clustering of DNA methylation profiles revealed separation into three groups: designated group 1 composed of clear cell and papillary meningiomas, whereas group 2A comprised predominantly atypical meningiomas and group 2B enriched for rare high-grade subtypes (rhabdoid, chordoid). Meningiomas from NF2 patients clustered exclusively within groups 1 and 2A. When compared with a dataset of 105 adult meningiomas, the pediatric meningiomas largely grouped separately. Targeted panel DNA sequencing of 34 tumors revealed frequent *NF2* alterations, while other typical alterations found in adult non-*NF2* tumors were absent. These data demonstrate that pediatric meningiomas are characterized by molecular features distinct from adult tumors.

**Supplementary Information:**

The online version contains supplementary material available at 10.1007/s00401-021-02351-x.

## Introduction

Among adult patients, meningiomas represent the most common primary intracranial tumor, accounting for 36.6% of newly diagnosed primary brain tumors [17]. In contrast, meningiomas are rare among children and young adults, accounting for only 3–5% of intracranial neoplasms [16, 17].

Prior clinical and genetic studies have suggested that differences exist between pediatric and adult meningiomas in regard to anatomical site and clinical behavior [20, 21, 26]. While the vast majority of adult patients with meningiomas presents with sporadic tumors, pediatric patients are more commonly affected by an underlying tumor predisposition syndrome, mainly NF2. One study showed that pediatric meningiomas harbored *NF2* deletions frequently (82%) along with more aggressive histological features (high mitotic count, brain invasion) [21]. The same study reported much higher frequencies of deletions in DAL-1, 1p, and 14q compared to adult counterparts. Recently it was reported that some pediatric meningiomas may carry YAP1 fusions [35].

Due to the significant risk of underlying NF2, young patients presenting with a solitary meningioma should be referred for human genetic counseling focused on NF2 surveillance including genetic testing [19] Besides sporadic and NF2-related meningiomas, radiation induced meningiomas represent another clinically distinct subset [32].

The knowledge regarding the molecular alterations in adult meningiomas has been increased substantially over the last 5 years. While the fundamental role of biallelic *NF2* loss in meningiomas from NF2 patients and approximately 50% of sporadic meningiomas has long been recognized [27, 28], a spectrum of recurrent oncogenic driver mutations including in *SMO*, *KLF4*, *TRAF7*, *AKT1*, or *PIK3CA* has more recently been identified, and are mutually exclusive with *NF2* alterations. Clinically relevant associations between driver mutations, anatomical site and histological subtype have been recognized, and are reviewed in [23]. Some of these mutations such as in the *SMO* or *AKT1* genes are now considered for molecular targeted therapies in case of recurrent or inoperable disease [23]. Similar to meningiomas arising in the setting of germline *NF2* mutations, radiation induced meningiomas are characterized by *NF2* inactivation [1]. Recently, based on methylation profiling six different subgroups of meningiomas in adults have been introduced, which differ by site, histological subtype, and prognosis [31].

In contrast, studies addressing the molecular alterations in pediatric meningiomas by comprehensive high-throughput analyses are rare. One study failed to detect the commonly observed mutations in *AKT1*, *SMO*, *KLF4*, and *TRAF7* (exon 17) by using conventional Sanger sequencing [3], and further comprehensive studies have not been published. Here we report the results from a comprehensive analysis of 37 pediatric meningiomas, integrating clinicopathological with genomic data derived from DNA methylation profiling and targeted panel sequencing.

## Materials and methods

### Tumor material

We analyzed 41 samples from 37 patients with meningioma (age range: 1–21 years, median age: 10 years). The study was approved by the local institutional review board of the University Hospital Magdeburg (#19/14). For 32 patients, only formalin-fixed paraffin-embedded (FFPE) samples were available for histological analysis. Tumors were graded according to the current 2016 WHO classification of brain tumors [14]. Ki-67 proliferation index of the tumors was assessed immunohistochemically using standard procedures. Tumor localization was stratified into convexity, skull base, spinal, and intraventricular sites. Clinical information regarding NF2 status was available for 32 patients, seven of which met clinical diagnostic criteria for NF2. Information regarding tumor recurrence was available for 17 patients. In four patients, prior CNS irradiation for tumor therapy (retinoblastoma, medulloblastoma, ependymoma, ALL) suggests that the meningiomas are secondary neoplasms.

### Immunohistochemistry [tissue microarray (TMA)]

From a total of 19 cases, we generated two TMAs as previously described [18]. The following antibodies were subjected to TMA samples: Akt/phospho-Akt, MAPK/phospho-MAPK, mTOR, phospho-p70S6K, 4EBP/phospho-4EBP, PDGF receptor, p38MAPK, TGFβR2, E-Cadherin, and MMP9 (all from Cell Signaling, MA).

### Sanger sequencing

DNA extracted from FFPE tissue was processed using standard procedures as previously described [42]. We analyzed all meningiomas for known hotspot mutations in the genes *SMO* (L412F and W535L) [4], *AKT1* (E17K) [42], and *KLF4* (K409Q) [41].

### 450 K methylation profiling

For methylation analysis and copy-number analysis of the samples, Illumina 450 k Human BeadChip (Illumina, San Diego, CA, USA) analyses were performed as previously described [38].

### Panel sequencing analysis

Panel sequencing for 130 genes reported to be mutant in meningioma based on a literature search done in October, 2014 was done by applying a custom hybrid-capture approach (Agilent Technologies, CA, USA) as described previously [29]. The panel was designed to assess the frequency of known mutations in the respective methylation classes and did not aim to detect novel mutational events.

### Whole genome sequencing

DNA libraries were prepared from tumor and matched normal tissue (blood) available for five pediatric meningioma cases (three with recurrent tumor samples available) and sequenced on three lanes each on HiSeq2000 instruments (2 × 100 bp). DNA libraries were prepared according to the Illumina TruSeq Nano DNA Library protocol using the TruSeq DNA Nano kit (Illumina, Hayward, CA) and sequenced on one lane on HiSeq X (2 × 151 bp) using the HiSeq X Ten Reagent Kit v2.5 (both Illumina).

### Alignment and detection of small variants

The raw reads were mapped to the human reference genome (build 37, version hs37d5), using BWA mem (version 0.7.15, with parameter −*T* 0), sorted using SAMtools (version 0.1.19), and duplicate reads were marked using Sambamba (version 0.6.5, with parameter −*t* 1 −l 0 –hash-table-size = 2,000,000 –overflow-list-size = 1,000,000 –io-buffer-size = 64). Using the tumor and corresponding matched normal samples, somatic small variants (SNVs and indels) were called using the in-house pipelines as described earlier [32]. Briefly, somatic SNVs were called using SAMtools mpileup (version 0.1.19, with parameters −REI −q 30 −ug) and bcftools on tumor sample and then queried in the control samples (with parameters −ABRI −Q 0 −q 1). To enable calling of variants with low allele frequency we disabled the Bayesian model in bcftools (by setting −p 2). The raw calls were annotated with ANNOVAR and many publicly available tracks such as 1000 Genome variants, single nucleotide polymorphism database (dbSNP), genomic repeat and low complexity regions and locally available controls. Confidence scores for these variants were annotated as described previously [11]. Indels were called using Platypus (version 0.8.1, with parameters −bufferSize = 100,000 −maxReads = 5,000,000), and were annotated similar to the somatic SNVs. High confidence somatic indels were required to have the genotype 0/0 (homozygous to reference allele) in the control sample and the platypus filter tag ‘PASS’ or, to enable the detection of somatic variants with low allele frequency, pass custom filters when Platypus reported ‘allele bias’.

Somatic small variants misclassified as germline due to contamination of the matched normal samples with tumor DNA/cells were rescued using the in-house tool TiNDA (Tumor in Normal Detection Analysis). VAFs from variants which are classified as ‘germline’ (i.e. variant reads have been identified in both tumor and the matched normal sample), and which are novel or rare (minor allele frequency (MAF) < 0.001 in gnomAD (version 2.0.1) and not present in the above local control of 280 WGS samples) were clustered using EM-based unsupervised clustering from Canopy (version 1.2.0) [10]. Clusters in which at least 85% of the members have a tumor variant allele frequency (VAF) of at least 0.01 and a matched control VAF below 0.45 were considered as misclassified somatic variants. These rescued somatic SNVs and indels were mapped to the mpileup and Platypus raw calls and variants with confidence score > 7 were merged into the final somatic small variant calls. Variants in the remaining clusters were classified as rare germline and were annotated as rare high confidence germline variants if they had a confidence score > 7 in the corresponding raw calls.

### Structural variant detection

Genomic structural variants were detected using SOPHIA (version 34.0; https://bitbucket.org/utoprak/sophia) as described earlier [32], using a background population database consisting of 3261 WGS controls across different diseases (published TCGA cohorts and published/unpublished DKFZ cohorts) and sequencing technologies (100 bp read length Illumina HiSeq 2000/2500 and 151 bp read length Illumina HiSeq X) aligned using the same alignment settings and workflow as used in the present study. Gencode V19 was used for the gene annotations.

Copy number states were called and tumor purity and ploidy were estimated using ACEseq (Allele-specific copy-number estimation from sequencing; https://www.biorxiv.org/content/early/2017/10/29/210807) as described previously [32]. In cases where ACEseq provides multiple purity and ploidy solutions, the lowest ploidy solution which allowed to fit the majority of genomic segments to integer copy numbers and which was consistent with the mutant allele frequency distribution of somatic SNVs was manually selected.

### Statistical analyses

Statistical analyses were performed using IBM SPSS Statistics, version 25 (IBM Corporation and its licensors, 1989, 2017) with a nominal error level of 5%. According to the type of variables (categorial, metrical or time-to-event endpoints), comparisons were carried out in chi-square, Mann–Whitney or log rank tests. For pairwise comparisons with three groups, the closed testing procedure was used (global test followed by pairwise comparisons) in categorial and time-to-event data and the Kruskal–Wallis tests including adjusted pairwise tests for metrical data. However, due to the limited number of available cases and the exploratory aim of the analyses, no adjustment for considering multiple endpoints has been made.

For unsupervised hierarchical clustering of 37 histologically defined pediatric meningioma and 105 reference samples, we selected the 10,000 most variably methylated CpG sites across the dataset according to median absolute deviation. Pairwise similarity of samples was calculated using Euclidean distance. Clusters were then linked according to the Ward’s linkage method.

## Results

**Fig. 1:**
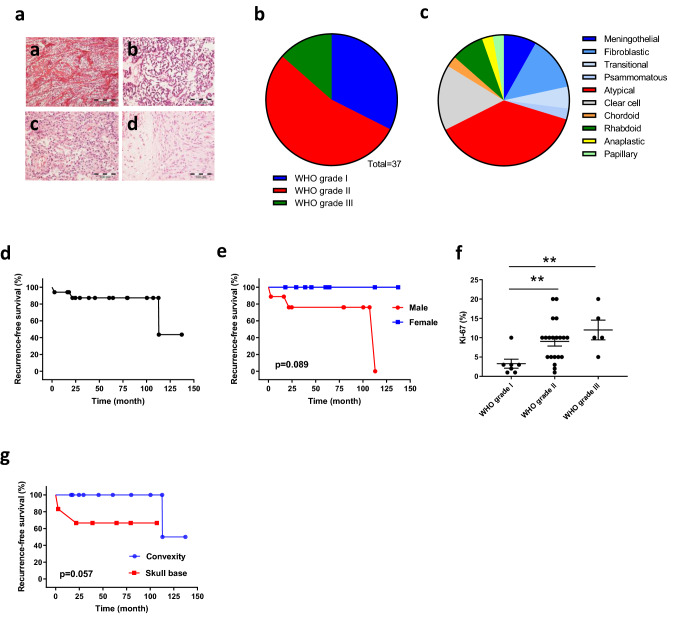
Pediatric meningiomas are aggressive tumors with uncommon histological features. **a** Examples of histological subtypes more frequent among pediatric meningiomas than in adult tumors. **a** Clear cell meningioma WHO grade II. **b** Chordoid meningioma WHO grade II. **c** Rhabdoid meningioma WHO grade III. **d** Atypical meningioma WHO grade II with brain invasion. Distribution by WHO grade (**b**) and histological variants (**c**). **d**, **e** Kaplan–Meier curves showing clinical characteristics of 17 pediatric meningiomas with follow-up data available. **f** Proliferation activity as determined by Ki-67 immunostaining within different WHO grades. **g** Recurrence-free survival according to the tumor localization (skull base or convexity)

The histological subtype distribution among the 37 primary pediatric meningiomas which were graded according to the 2016 WHO classification [14] of brain tumors is shown in Fig. 1a–c. While 30% of tumors were of WHO grade I, the largest group consisted of atypical WHO grade II meningiomas (57%). Among 5 WHO grade III meningiomas (14%), three tumors were rhabdoid meningiomas. Thus, pediatric meningiomas are predominantly characterized by a more aggressive histology than WHO grade I, which is in contrast to their adult counterparts [23]. This was reflected by a relatively high proliferation activity, with a mean Ki-67 proliferation index of 8.3% (1–20%) and a mean number of mitoses per 10 HPF of 1.9 (0–20). Brain invasion was seen in 6/32 samples analyzed (16.2%), and tumor necrosis was present in 10/32 cases (27%). The cohort contained slightly more male (*N* = 20) than female patients (*N* = 17). In Fig. 1d, the time to tumor recurrence for patients with available follow-up information is shown. As shown in Fig. 1e, male patients had a trend to less favorable outcome than female patients (*p* = 0.089). Proliferation activity as counted by Ki-67 staining was significantly higher in WHO grade II and grade III tumors than in WHO grade I meningiomas (Fig. 1f). Except one patient with spinal meningioma (2.7%), 36 tumors (97.3%) were located intracranially. Most tumors were found at the convexity (*N* = 18, 48.6%), followed by skull base (*N* = 15, 40.5%) and ventricular system (*N* = 3, 8.1%). Skull base localization was associated with slightly less favorable prognosis compared to convexity localization (*p* = 0.057, Fig. 1g).

### Somatic and germline NF2 alterations are the dominating molecular event in pediatric meningiomas

In an initial attempt, we wondered whether recurrent somatic mutations reported to occur in adult meningiomas exclusive of *NF2* alterations are present in pediatric meningiomas as well [5, 7, 25]. However, by Sanger sequencing, we did not detect mutations at the established hotspots in *AKT1*, *SMO*, *KLF4* and *TRAF7*, suggesting different tumor drivers acting in this age group. This observation prompted us to further analyze the samples by WGS, DNA methylation profiling, and panel sequencing.

**Fig. 2:**
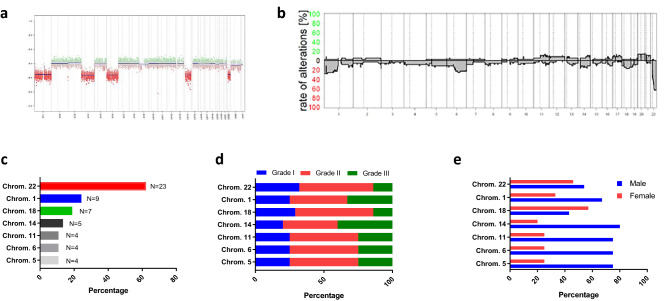
Summary of copy-number variations (CNV) in pediatric meningiomas derived from methylation analysis. **a** Representative copy-number plot. **b** Summary of CNV among all 37 pediatric meningiomas. Frequency of the most common CNV (**c**), and distribution of CNV by WHO grade (**d**) and sex (**e**)

First, we subjected all 37 patient samples to methylation profiling and DNA copy-number analysis. The most frequent alteration was loss of chromosome 22 including the *NF2* gene (*N* = 23, 62.2%), followed by loss of chromosomal material on chromosome 1 (*N* = 9, 24.3%), chromosome 18 (*N* = 7, 18.9%), and chromosome 14 (*N* = 5, 13.5%) (Fig. 2a–c). The distribution among the WHO grades, as well as between males and females is shown in Fig. 2d, e.

**Fig. 3:**
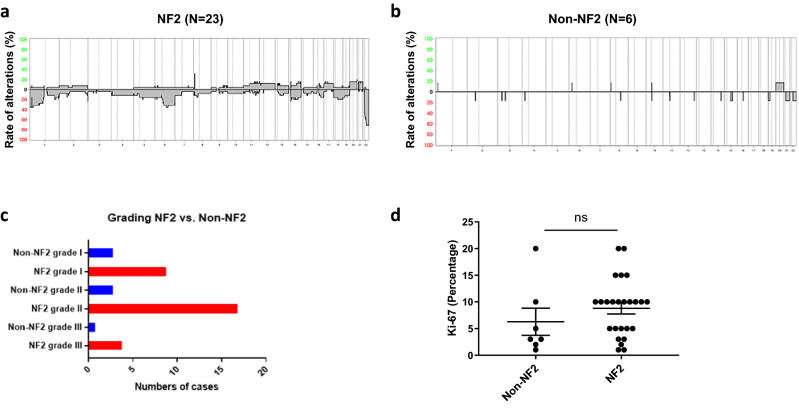
Comparison of pediatric meningiomas with (*NF2*) or without (non-*NF2*) allelic losses at chromosome 22. Summary of CNV in cases with (**a**) or without (**b**) *NF2*-LOH. **c** Distribution among WHO grades. **d** Proliferation activity (Ki-67 labeling)

Because loss of chromosome 22 turned out to be the most frequent molecular event in our series, tumors with (NF2) or without (Non-NF2) alterations were compared (Fig. 3). Comparing the CNV profile between both groups, *NF2*-altered tumors (Fig. 3a) had a higher rate of CNVs than Non-*NF2* tumors (Fig. 3b), irrespective of WHO grade (Fig. 3c). The highest frequency of CNVs among *NF2*-altered tumors was found in the WHO grade II group. *NF2*-altered tumors tend to have higher proliferation activity (Fig. 3d). In contrast to chromosomal losses, gain of chromosomal material was seen infrequently. Gain of chromosome 11 (*p* = 0.042) and chromosome 21 (*p* = 0.015) were independently associated with higher tumor grading. The presence of tumor necrosis was significantly associated with gain of chromosome 13 (*p* = 0.024). The histological variant was significantly associated with gain of chromosome 12 (*p* = 0.034), showing gains in one anaplastic, one atypical, and one chordoid meningioma, respectively. In line with this, gain of chromosome 12 was additionally associated with increased number of mitoses (*p* = 0.09). Gain of chromosome 14 was associated with higher mitotic activity (*p* = 0.05) as well. All tumors showed an unmethylated *MGMT* promoter.

Whole genome sequencing could be performed for a total of five meningiomas. Three of them showed germline *NF2* alterations together with somatic loss of the second allele, while two cases showed somatic *NF2* mutations. From three cases (case #3 & #8 with germline *NF2* alteration, case #6 with somatic *NF2* alteration), primary and recurrent tumors were available for WGS as well. As shown in supplementary Figure S2, recurrent tumors were similar to the primary tumors with the exception that the second and third recurrence from tumor #8, as well as recurrence from tumor #3 had an additional amplification of the *LZTR1* gene.

### Pediatric meningiomas form three distinct methylation groups

Next we wondered whether separate methylation groups are present among pediatric meningiomas. Applying a methylation profile-based brain tumor classifier [6], 35/37 (95%) samples were classified as meningioma. However, one case was classified as high-grade glioma but showed histologically features of a papillary meningioma. Another case, meningioma by histology as well, was classified as a desmoplastic infantile ganglioglioma [DIGG]. Interestingly, a TSNE plot (Figure S6) revealed that the tumor classified as DIGG grouped with other meningiomas (blue arrow), while the papillary meningioma indeed fell into the high-grade glioma group (green arrow).

**Fig. 4:**
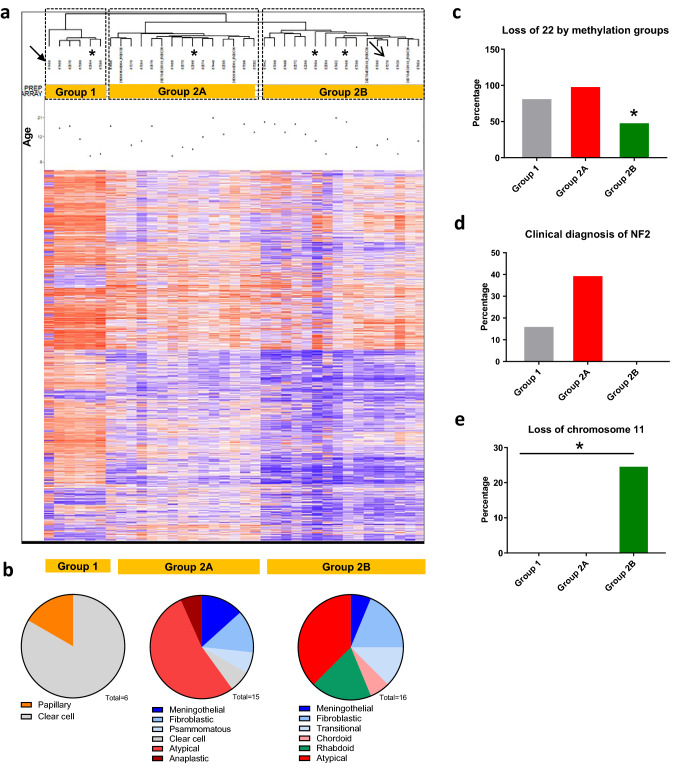
Methylation profile of 37 pediatric meningiomas. **a** Unsupervised hierarchical clustering reveals three subgroups (1, 2A, 2B). Arrows indicate tumors not classified as meningioma in the meningioma classifier for adult tumors. Radiation-induced pediatric meningiomas are marked by (*). **b** Relation between histological variant and methylation subgroup. Frequency of *NF2*-LOH (**c**) and clinical diagnosis of NF2 (**d**) among the three subgroups. **e** Frequency of LOH at chromosome 11 among the three subgroups. Note that c-e display data for 35 cases after exclusion of two cases (arrows) which were not classified as meningioma using the meningioma classifier [31]

Unsupervised hierarchical clustering of all 37 pediatric meningiomas revealed separation into three subgroups (Fig. 4a). On the far left, a group of six patients could be distinguished (group 1). Among the remaining 31 cases, two major subgroups were formed, consisting of 15 (group 2A) and 16 (group 2B) cases. Group 1 was comprised of the single papillary meningioma within the series (arrow) and five clear-cell meningiomas. Group 2A with 15 cases consisted mainly of atypical meningiomas (*N* = 8), one clear-cell meningioma, one anaplastic, as well as 5 WHO grade I tumors of meningothelial, fibroblastic, transitional, and psammomatous variant. Group 2B comprising 16 tumors covered 6 WHO grade I tumors, three rhabdoid meningiomas, one chordoid meningioma, and six atypical meningiomas (Fig. 4b). The recurrent tumors which had been undergone WGS grouped into group 2A.

The methylation profile-based separation into three subgroups prompted further comparisons between the proposed methylation subgroups 1, 2A and 2B. Loss of chromosome 22 was significantly less frequent in group 2B (Fig. 4c). If the groups were compared regarding the clinical information about signs of NF2, no patient from group 2B had been diagnosed clinically for this disease (Fig. 4d). These findings suggest that group 1 and 2A but not group 2B are driven by *NF2* alterations. Additionally, group 2B showed frequent losses of chromosome 11 (Fig. 4e), while group 1 was characterized by loss of chromosome 19. The proliferation activity, however, was not significantly different between the three groups (supplementary Figure S1).

### Panel sequencing

**Fig. 5:**
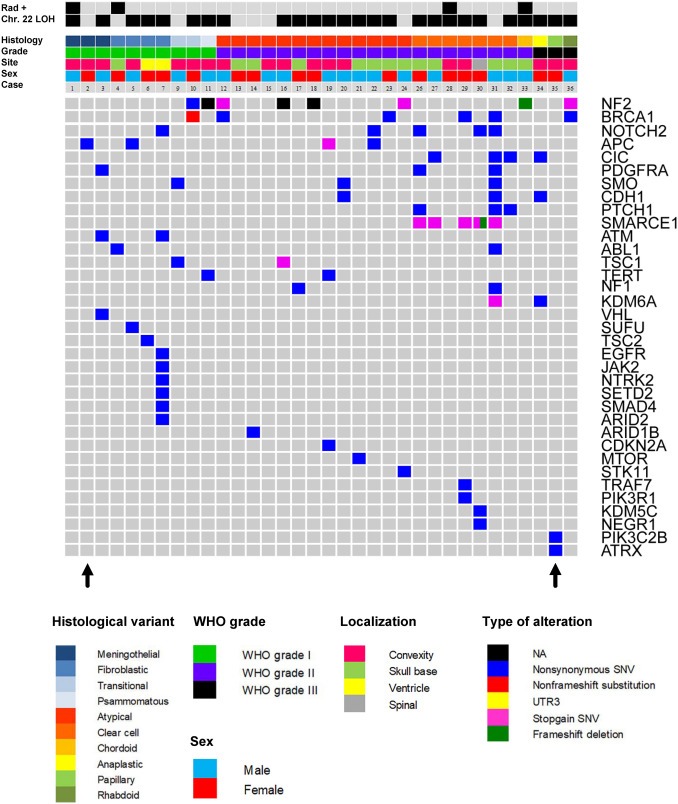
Oncoplot summarizing relation between histological variant, WHO grade, sex, tumor localization (site), prior irradiation, and mutational spectrum derived from panel sequencing of 130 genes. 34 cases were analyzed; for three cases no sufficient material was available. Arrows indicate the two samples from Fig. 4a which were not classified as meningioma

Because we initially did not detect characteristic somatic mutations known from adult meningiomas by Sanger sequencing, our cohort was further screened for mutations in 130 genes by panel sequencing as previously reported [30]. The oncoplot in Fig. 5 shows the results from 34 cases with sufficient material for analysis, together with clinicopathological data. As already suggested by the methylation profiling and respective copy-number data, the most frequent alteration affected the *NF2* gene (8/34, 24%). Additional alterations were occasionally found in *BRCA1*, *RGPD3*, *APC*, *TSC1*, and *KDM6A* with uncertain relevance and unknown germline status. All *SMARCE1* alterations were present in clear-cell meningiomas (mutations in 5/7 tumors detected). No hotspot mutations found in adult meningiomas were detected for *SMO*, *KLF4*, and *AKT1*. Moreover, no *TERT* promoter mutations were detected. Additional immunohistochemistry for BAP1 in two of the three rhabdoid meningiomas showed retained immunoexpression, suggesting absence of BAP1 mutations (Figure S5).

### Methylation profiles differ between adult and pediatric meningiomas

**Fig. 6:**
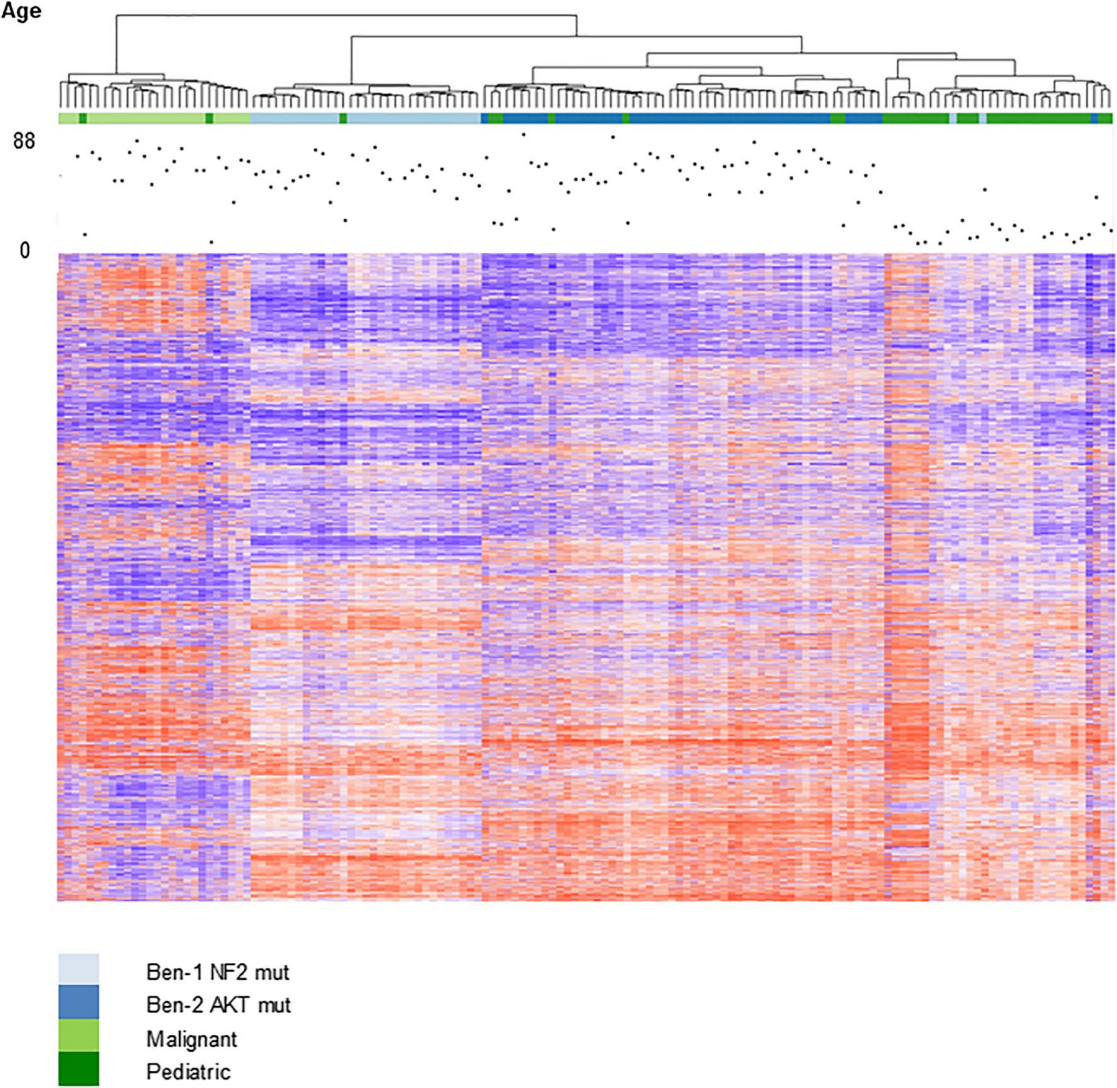
Unsupervised hierarchical clustering of 105 adult and 37 pediatric meningiomas. Ben-1 and Ben-2 refers to methylation subgroups of adult meningiomas proposed by Sahm et al. [31]

Next, we analyzed the methylation profiles from all 37 pediatric meningiomas together with a group of 105 adult meningiomas which has been described in detail before [31]. As shown in Fig. 6, the vast majority of pediatric meningiomas (*N* = 28) again clustered into a separate group. Moreover, the three subgroups (1, 2A, 2B) defined above could be distinguished again. The few pediatric tumors clustering with adult malignant (light green) or benign *NF2*-mutated tumors (light blue) were an anaplastic meningioma, an atypical meningioma, and a fibroblastic meningioma.

### Signaling pathways activated in pediatric meningiomas

**Fig. 7:**
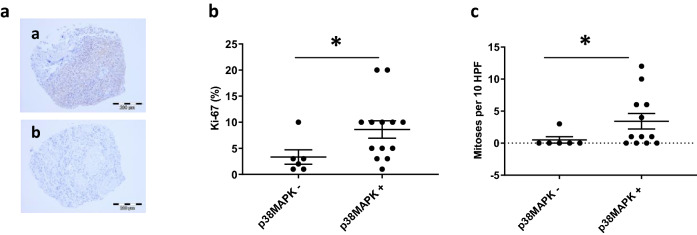
Analysis of p38MAPK signaling in pediatric meningiomas. Examples of p38MAPK-immunopositive (**a**) and immunonegative (**b**) tumor samples derived from a tissue micro array (TMA). Pediatric meningiomas with activation of p38MAPK have higher proliferation activity (**b**) and mitotic count (**c**) compared to meningiomas without p38MAPK expression

To evaluate potential treatment targets in our cohort, we performed immunohistochemical analyses for several proteins involved in essential cellular signaling pathways. A TMA containing 20 pediatric meningiomas was studied by immunohistochemistry. While for most of the proteins the presence or absence of expression was unrelated to clinical parameters or detected molecular alterations, we found that the expression of p38MAPK was significantly associated with increased mitotic activity (*p* = 0.036) and increased Ki67 proliferation index (*p* = 0.041) (Fig. 7a–c).

## Discussion

We have performed comprehensive molecular analysis of a large group of pediatric meningiomas, revealing that these tumors are substantially different from their adult counterparts by means of histological subtype distribution, methylation profiles, and mutational landscape.

While meningiomas are the most frequent primary intracranial tumors in adults, they are exceptionally rare among children [16] and adolescents [17]. It had been already reported that the gender distribution is different in pediatric meningiomas, with more male patients affected, and that the spectrum of histological variants is different [21]. These epidemiological data were confirmed in our series. A meta-analysis compiling data from 677 pediatric and adolescent meningioma patients showed that patients with NF2 had worse recurrence-free survival (RFS) time than patients without [12]. They also found reduced RFS in patients with grade III meningiomas, compared to grade I/II tumors. However, we did not see a clear reduction of the time to tumor recurrence in grade III meningiomas, although the number of cases with follow-up data available was rather small. In contrast, grade III meningiomas showed similar time to tumor recurrence as grade I meningiomas. This might be explained by the fact that the group of grade III tumors beside anaplastic meningiomas included rare meningioma variants like papillary or rhabdoid meningiomas, for which follow-up data from larger series of pediatric meningiomas are not available.

One of the major findings is the high frequency of chromosome 22 alterations in our cohort. Alterations in the tumor suppressor gene *NF2* located on chromosome 22q are the most common finding in meningiomas occuring in adults. Loss of heterozygosity (LOH) and inactivating *NF2* mutations are found in 40–80%, supporting a classical two-hit hypothesis for meningioma development [27, 28]. The alterations include deletions, insertions, and mutations affecting splice sites, resulting in a non-functional protein [13]. For pediatric meningiomas, Perry et al. have reported a detailed analysis on *NF2* in this patient group, with *NF2* deletions in 86% of NF2 patients and 70% in non-NF2 patients [21]. We found LOH on chromosome 22 in 76% of samples, confirming the dominating role of this tumor suppressor gene in pediatric meningiomas.

Recently, a number of Non-*NF2* alterations have been identified in sporadic adult meningiomas (reviewed in [23]). The most frequent one affects the *TRAF7* (TNF receptor associated factor) gene [7]. Less frequently, alterations in *SMO*, *KLF4*, *AKT1*, *PIK3CA* are detectable. In our initial Sanger sequencing approach we did not detect any of these alterations, which is in line with recent data from Battu et al. [3]. Recently, Toland et al. reported similar findings with absence of the non-*NF2* alterations in pediatric meningiomas [39]. Together with the high frequency of *NF2* alterations it can be concluded that pediatric meningiomas are mainly driven by loss of this tumor suppressor.

*TERT* promoter mutations have been found in about 6% of adult meningiomas and were associated with higher meningioma grade and early recurrence [30, 37]. Surprisingly, despite the high frequency of aggressive meningioma subtypes, especially atypical meningiomas, no *TERT* promoter mutations were detected.

The most intriguing finding of our study was the observation that unsupervised clustering of the methylation profile revealed three novel subgroups. Analysis of DNA methylation to characterize brain tumors has been recently introduced in molecular neuropathology as a valuable tool not only to support conventional histological diagnoses, but also to uncover biological relations between morphologically unrelated tumors [6]. For adult meningiomas, Sahm et al. have recently established a methylation-based classification system, separating six different methylation classes with distinct molecular and clinical characteristics [1]. The current study expands this methylation-based classification to pediatric meningiomas, suggesting three distinct subgroups with separate molecular characteristics.

One group (designated group 1) covering 6 pediatric meningiomas was almost exclusively built by clear-cell meningiomas. Clear cell meningiomas are known to be most prevalent in pediatric and adolescent meningiomas [24], and their molecular basis is defined by alterations of the *SMARCE1* tumor suppressor gene [8, 36]. Consistently, panel sequencing confirmed the presence of *SMARCE1* mutations in five of these tumors. In line with previous data, group 1 was additionally characterized by allelic losses at chromosome 22. Another intriguing molecular feature of group 1 was the high frequency of allelic losses at chromosome 19. This aberration has been rarely described in meningiomas in association with high-grade meningiomas [2, 15].

The remaining 31 pediatric meningiomas formed a large group which could be separated into two distinct subgroups designated as group 2A and 2B. Group 2A was dominated by atypical meningiomas WHO grade II. Interestingly, in this group 100% of cases showed chromosome 22 loss, as well as the highest frequency of clinically diagnosed NF2. Thus, this group can be described as the *NF2*-driven subgroup of pediatric meningiomas.

The third group, designated group 2B, was characterized by a mixture of histological variants but included all rhabdoid meningiomas WHO grade III. Moreover, only in this group allelic loss of chromosome 11 were observed, while loss of chromosome 22 was rare and clinical signs of NF2 were absent. Meningiomas with rhabdoid features were initially thought to be characterized by aggressive biology [22]. Recently it could be shown that the clinical course of rhabdoid meningiomas largely depends on the loss of the tumor suppressor *BAP1*, irrespective of the underlying rhabdoid phenotype [33, 34, 40].

*YAP1* fusions have been recently reported among pediatric meningiomas [35]. However, chromosomal rearrangement around the *YAP1* locus serving as a surrogate for *YAP1* alterations were not detected in our cohort, while RNA sequening would be required to address this question appropriately.

Another striking observation is that the methylation profile of pediatric meningiomas differs clearly from the one observed in adult meningioma patients. By analyzing pediatric and adult tumors together the pediatric group still was clearly separated. Despite the high frequency of *NF2* alterations in the pediatric group, the tumors did not fall into the cluster of *NF2*-altered adult meningiomas. This indicates that the underlying biology is substantially different between pediatric and adult meningiomas.

The lack of NF2 patients being represented in the 2B methylation cluster could have clinical implications. While approximately 40% of young patients presenting with a solitary meningioma have an underlying germline NF2 mutation, the diagnosis can be difficult to ascertain genetically due to mosaic status and may require years to confirm clinically [19]. Moreover, in our retrospective series it appeared difficult to trace back clinical features according to the consensus criteria [9]. If our findings can be confirmed in a larger cohort of NF2 meningioma patients, methylation profiling could provide a valuable diagnostic adjunct.

Taken together, our data show that pediatric meningiomas can be separated into groups with distinct morphological and molecular features, and that pediatric meningiomas are distinct from adult counterparts despite sharing a high frequency of *NF2* alterations. However, compared with adult counterparts and the well-established methylation classes with clear prognostic implications [31], for pediatric meningiomas the establishment of prognostic groups based on the methylation classification appears less likely, given the low frequency of meningiomas in this age group.

## Supplementary Information

Below is the link to the electronic supplementary material.

Supplementary file1 **S1** Proliferation activity by methylation subgroups. **S2** Oncoplot for 3 recurrent pediatric meningiomas (WGS analyses). **S3** Recurrence-free survival by WHO grading. **S4** Recurrence-free survival by NF2. **S5** BAP2 staining for two out of three rhabdoid meningiomas showing retained immunoexpression (TIF 1789 KB)

Supplementary file2 (TIF 745 KB)
