# Supplementary figures and images for: Molecular profiling of pediatric meningiomas shows tumor characteristics distinct from adult meningiomas
Source: Acta Neuropathol. 2021 Sep 8;142(5):873–86. doi: 10.1007/s00401-021-02351-x (PMC8500891; doi:10.1007/s00401-021-02351-x)

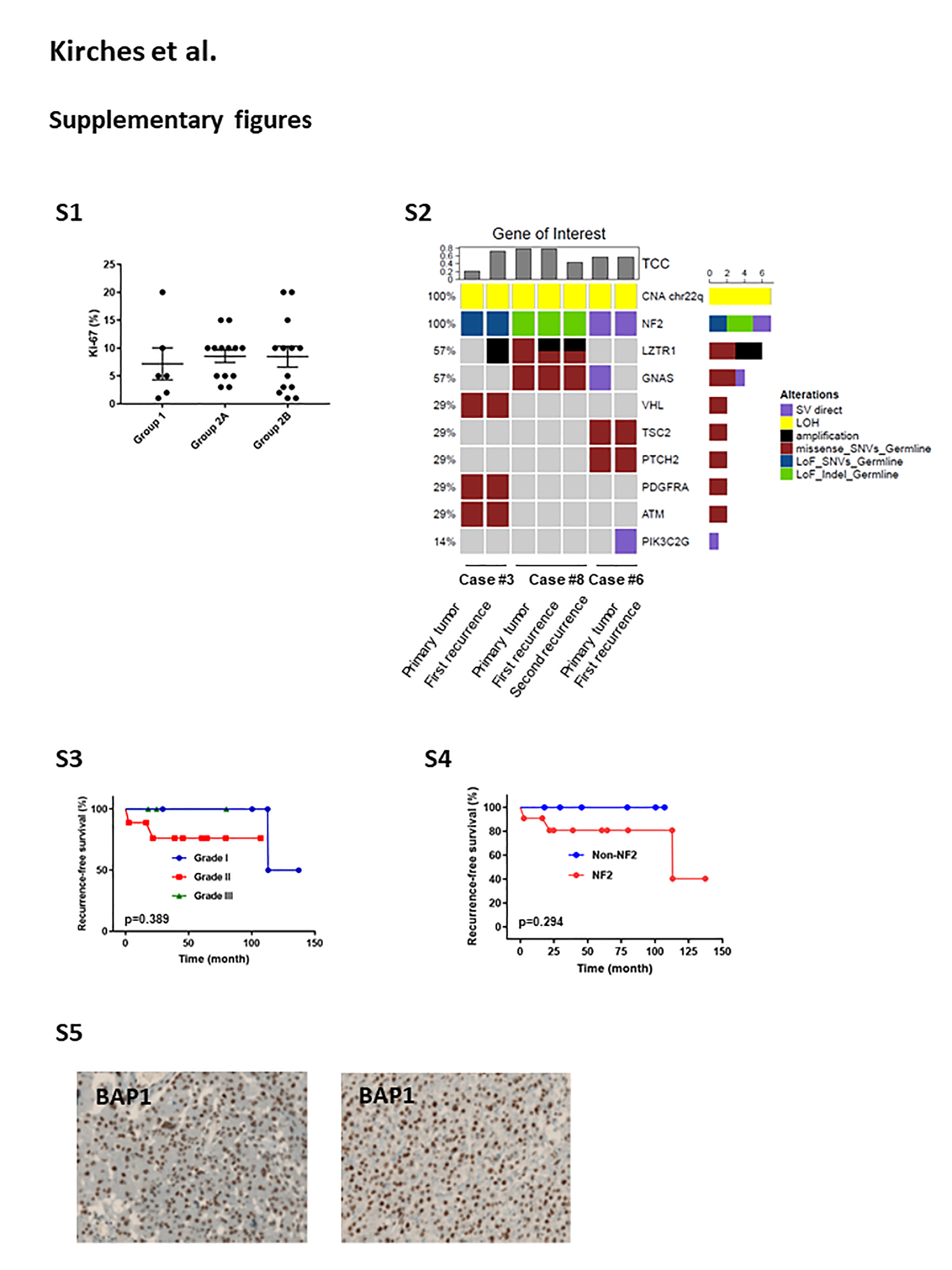

Supplement: Supplementary file 1 — Supplementary file1 S1 Proliferation activity by methylation subgroups. S2 Oncoplot for 3 recurrent pediatric meningiomas (WGS analyses). S3 Recurrence-free survival by WHO grading. S4 Recurrence-free survival by NF2. S5 BAP2 staining for two out of three rhabdoid meningiomas showing retained immunoexpression (TIF 1789 KB) [file 401_2021_2351_MOESM1_ESM.tif]

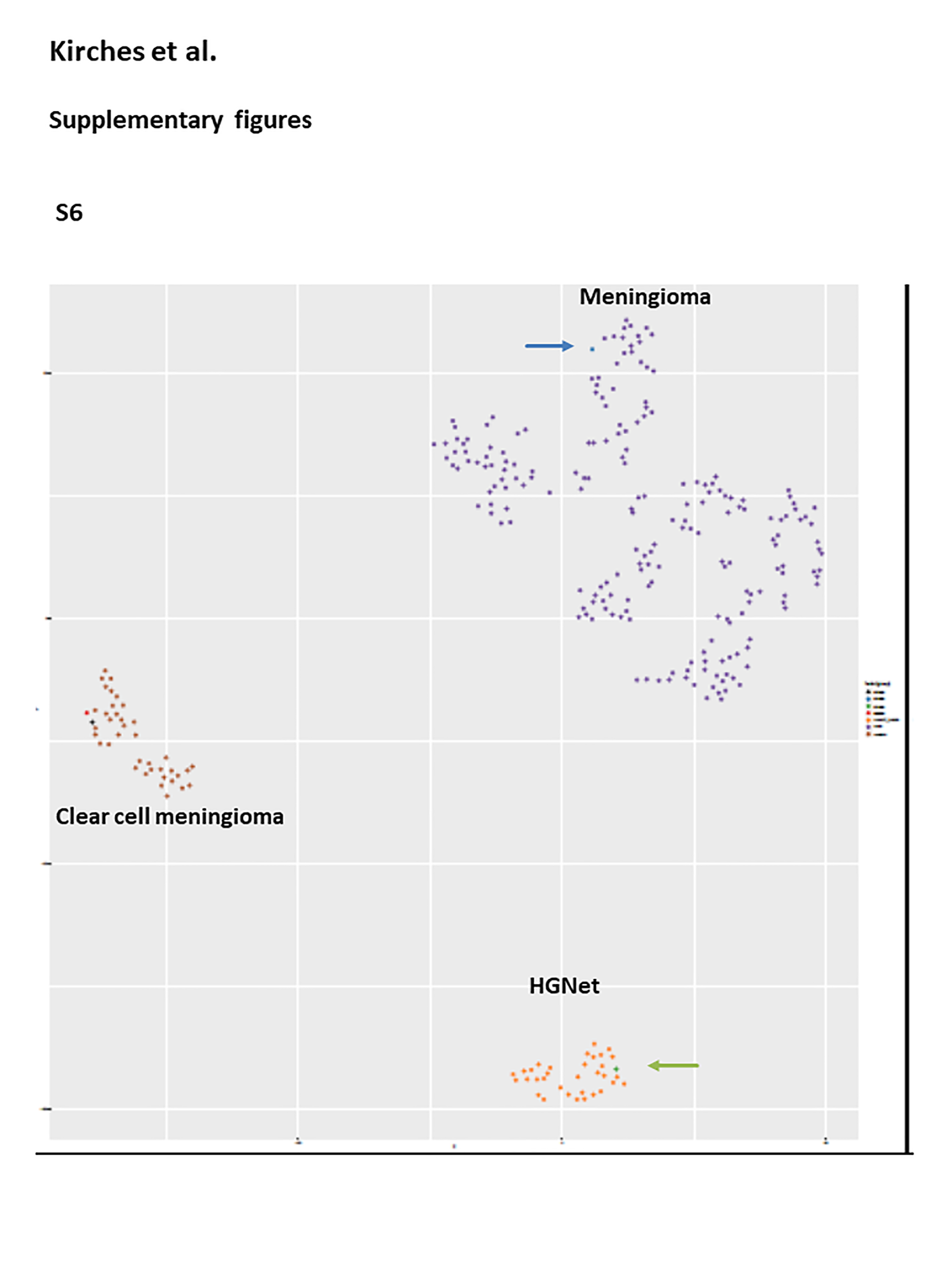

Supplement: Supplementary file 2 — Supplementary file2 (TIF 745 KB) [file 401_2021_2351_MOESM2_ESM.tif]
